# Polypeptides-Based Nanocarriers in Tumor Therapy

**DOI:** 10.3390/pharmaceutics16091192

**Published:** 2024-09-10

**Authors:** Juhua You, Yifei Guo, Zhengqi Dong

**Affiliations:** 1 School of Pharmacy, Heilongjiang University of Chinese Medicine, No. 24, Heping Road, Xiangfang District, Harbin 150040, China; 2 Institute of Medicinal Plant Development, Chinese Academy of Medical Sciences & Peking Union Medical College, No. 151, Malianwa North Road, Haidian District, Beijing 100193, China

**Keywords:** polypeptides-based nanocarriers, drug delivery systems, antitumor, immunomodulation, challenge

## Abstract

Cancer remains a worldwide problem, and new treatment strategies are being actively developed. Peptides have the characteristics of good biocompatibility, strong targeting, functional diversity, modifiability, membrane permeable ability, and low immunogenicity, and they have been widely used to construct targeted drug delivery systems (DDSs). In addition, peptides, as endogenous substances, have a high affinity, which can not only regulate immune cells but also work synergistically with drugs to kill tumor cells, demonstrating significant potential for application. In this review, the latest progress of polypeptides-based nanocarriers in tumor therapy has been outlined, focusing on their applications in killing tumor cells and regulating immune cells. Additionally, peptides as carriers were found to primarily provide a transport function, which was also a subject of interest to us. At the end of the paper, the shortcomings in the construction of peptide nano-delivery system have been summarized, and possible solutions are proposed therein. The application of peptides provides a promising outlook for cancer treatment, and we hope this article can provide in-depth insights into possible future avenues of exploration.

## 1. Introduction

Cancer continues to be a significant threat to human health, with over 10 million new cases reported each year. However, mortality rates have declined in the past two years due to enhanced understanding of tumor biology and advancements in diagnostic equipment and treatments [1]. Traditional cancer treatment options encompass surgical resection, chemotherapy, and radiation therapy. Among these therapeutic methods, chemotherapy is widely employed, and a lot of chemotherapeutics are applied broadly in clinical settings, such as doxorubicin (DOX), paclitaxel (PTX), cyclophosphamide, vincristine, and so on. However, these chemotherapy drugs sometimes result in low therapeutic efficacy and severe adverse effects due to low bioavailability and multidrug resistance [2]. In addition, the metastasis of tumor cells usually occurs during the therapy process: it seems that only inhibiting tumor cells is insufficient to cure cancer.

**Figure 1:**
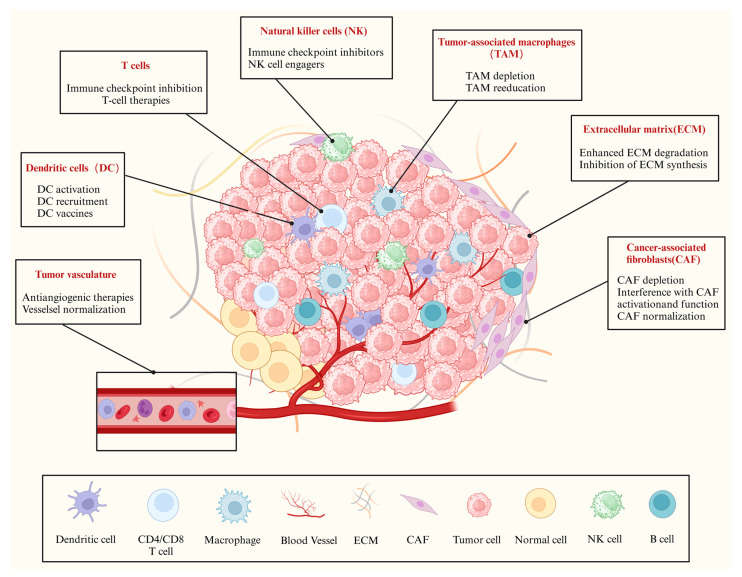
TME. The TME is defined as a complex and rich multicellular environment for tumor development. In general, the TME is mainly composed of many different types of components, including heterogeneous cancer cells; immune cells, such as T lymphocytes, B lymphocytes, tumor-associated macrophages (TAMs), dendritic cells (DC), and natural killer (NK) cells; interstitial cells, such as CAFs; and a network of blood and lymphatic vessels. Because each of these components is involved in a unique way in the regulation of tumor progression and treatment response, multiple treatments for the TME have been developed in recent years (listed separately in the figure). The figure has been prepared using BioRender, and a publication license was obtained.

With the study of the tumor microenvironment (TME) (Figure 1), it has been proven that tumor tissue involves a complex system: the system not only contains tumor cells, but also holds its own immune system. To treat tumors more effectively, immunotherapy has been developed. Immunotherapy mainly acts on immune cells and activates the body’s immune system by inhibiting immune negative regulators and enhancing the ability of immune cells to recognize antigens on the surface of tumor cells, thereby eliminating tumor cells [3]. Currently, there are three main types of tumor immunotherapy: immune checkpoint inhibitors (ICIs) therapy [4], adoptive cell therapy (TILs, TCR-T, and CAR-T) [5], and cancer vaccines [6]. Based on these therapies, commonly used small-molecule immune drugs mainly include resiquimod [7,8], nivolumab [9], pembrolizumab [10,11], atezolizumab [12,13], camrelizumab [14,15], and ipilimumab [16,17], which have made remarkable progress in activating toll-like receptors (TLRs) TLR7 and TLR8 receptors, blocking the PD-1/PD-L1 pathway, and opening a new era of tumor therapy. However, not only chemotherapeutic drugs but also immune drugs still present disadvantages hindering their application in clinical settings, such as the poor solubility of the drug, significant toxic side effects, and difficulty in successfully reaching the tumor site. Therefore, how to enhance the therapeutic efficacy of chemotherapy and immunotherapy is still a primary challenge.

To overcome the above problems, encapsulating drugs with materials to create DDSs have been considered as a potential technology for improving drug solubility, selectivity, and effectiveness [18,19]. This is mainly reflected in several aspects: (1) DDSs can increase the solubility of hydrophobic drugs to improve bioavailability. (2) DDSs can increase the accumulation of drugs at the tumor site due to the fact that they have an enhanced permeability and retention (EPR) effect, thereby improving the therapeutic effect. (3) Some nanomaterials present environmental responsive properties, such as enzyme-sensitive properties, thermal-sensitive properties, redox-sensitive properties, and pH-sensitive properties, which could be triggered by the microenvironment of tumor tissue to enhance the accumulation of drugs [20,21,22]. To construct effective DDSs, polymers (peptides and polysaccharides) [23,24], inorganic substances [25], polymer–lipid hybrids [26,27,28], liposomes [29,30], and dendrimers are utilized as nanocarriers [31,32]. Among these materials, peptides with potential clinical applications have been studied broadly in recent years.

Peptides are a class of compounds formed by covalently linking multiple amino acids through peptide bonds, which have unique physiological functions and play an important role in disease treatment. As of January 2024, over 180 peptide drugs have been approved for clinical use (some of these peptide drugs have been listed in Table 1). Peptides have excellent biocompatibility, high affinity, functional diversity, and simple synthesis modification as their advantages. One of the most significant advantages of peptides is their functional diversity compared to antibody–drug conjugates (ADCs), which allows them to perform multiple roles in drug delivery applications [33,34]. Firstly, peptides can self-assemble into well-defined structures due to their inherent aggregation capacity: this includes vesicles, rods, sheets, spirals, nanofibers, nanoparticles (NPs), and nanotubes [35]. These aggregates can then be utilized as therapeutic agent carriers for facilitating penetration [34]. The self-assembly structure of polypeptides is mainly caused by ionic bonds, hydrogen bonds, and hydrophobic interactions [36]. Secondly, some peptides are stimulus-responsive and able to respond to internal or external stimuli that enable controlled drug release. Thirdly, peptides can modify NPs with specific targeting capabilities that help them quickly internalize into particular cells. For tumor immunotherapy purposes, these properties of peptides are often used for enhancing antigen-to-antigen-presenting cells (APCs) delivery, stimulating T cells through artificial antigen presentation and increasing the levels of immune-stimulating cytokines in the TME [37,38,39,40], which have shown great potential for enhancing antitumor efficacy and stimulating robust antitumor immunity [40].

Here, a comprehensive review of the recent advances in peptide-based nanomaterials delivering drugs to kill tumor cells and regulate immune cells has been provided. Firstly, the antitumor progress of peptide-functionalized drug delivery vehicles (tumor-targeted peptides and stimulus-responsive peptides) and peptide drug delivery vectors is reviewed from the aspect of tumor cells. Secondly, from the perspective of immune cells, the role of peptide-based nanocarriers in regulating the three major systems (i.e., T cells, TAMs, and NK cells) is introduced. Thirdly, we give examples of peptides providing transport functions only as carriers. Finally, the advantages and limitations of peptide carriers in tumor treatment are discussed. Here, we mainly focus on the role of these polypeptides-based nanocarriers in tumor therapy.

## 2. Effect of Polypeptides-Based Nanocarriers on Tumor Cells

### 2.1. Peptide-Functionalized Drug Delivery Carriers

Multiple abnormal conditions in the TME stimulate rapid growth and the metastasis of tumor cells. Peptides with ideal drug delivery properties, for example, cell penetration and tumor targeting, have been coupled to all kinds of DDSs through coupling methods to rapidly facilitate the delivery of antineoplastic drugs. Based on the primary function of the peptide, this section mainly discusses tumor-targeting peptides and stimuli-responsive peptides.

#### 2.1.1. Tumor-Targeted Peptides

The high level of expression of certain molecules or receptors in tumors is closely related to malignant proliferation and metastasis. Based on receptor-mediated endocytosis, attached peptides enable DDSs to preferentially accumulate more in tumor tissues, tumor cells, and even subcellular organelles than in normal cells [58]. The following sections highlight examples of peptide-modified DSSs with tumor-targeting properties in the treatment of malignant tumors. The advantages and disadvantages of some peptides are listed in Table 2.

##### Tumor Cell-Targeting Peptides

This class of peptides refers to ligands that can specifically recognize and bind to tumor cell surface receptors. The drug carriers constructed from them can not only deliver drugs to specific sites, as well as reduce the poisonousness of drugs on normal cells, but they can also increase the accumulation of drugs. Among the various therapeutic approaches, tumor-targeted therapy has shown great promise as a precision therapy. With the continuous development of bacteriophage technology, more and more tumor cell-targeting peptides have appeared in the public’s field of vision, which provide a feasible means to kill tumor cells. In 1977, Erkki Ruoslahti’s team proposed the code hypothesis, that is, the existence of an identification system that guides cell localization, and they also proposed that code may be produced by perpetual gene rearrangements [77]. To do this, they isolated cell surface proteins that may mediate cell identification. After a series of studies, this cell surface protein was named fibronectin. Subsequently, cell attachment sequences, i.e., RGD sequences, were found in fibronectin. Years later, Robert Pytela isolated an RGD peptide-directed cell receptor (integrin) [78]. At present, it has been established that the RGD is a short peptide composed of Arginine-Glycine-Aspartic acid, which can specifically interact with integrins on the surface of tumor cells to achieve tumor targeting [79,80]. Several integrins (e.g., αvβ3, α5β1, etc.) are abundantly expressed in most tumor cells and in tumor microvasculature, and they play a role in mediating various cellular events [81]. Hence, nanomaterials modified with integrin ligands can obtain tumor cell targeting and enhanced endocytosis [79].

Currently, RGD can be divided into two types according to the different structure, including linear RGD and circular RGD (cRGD). When it interacts with the same type of integrin receptor, linear RGD exhibits lower affinity; this phenomenon could be explained by the linear RGD structure being more flexible and easier to form unexpected intramolecular hydrogen bonds with other residues. Because the linear RGD structure is more flexible, it is easy to interact with residues around the receptor and form intramolecular hydrogen bonds, thus making its stability in binding to integrin significantly weaker than that of cRGD [82]. In addition, linear RGDs are easily degraded by intracellular proteases due to their flexible structure; in contrast, cRGDs show higher stability and resistance to protease degradation [41]. Thus, cRGD is widely used as a ligand for specific integrin-mediated uptake. Tumor vascular endothelial cells highly express αvβ3 and αvβ5 integrin receptors, and efficient systemic antiangiogenic gene therapy was devised through tumor vascular targeting using cRGD-appended polyplex micelles that resulted in efficient antitumor therapy [83]. Dubey et al. prepared cRGD-modified albumin nanospheres loaded with fluorouracil for targeting tumor vasculature [81]. They have shown several advantages over conventional nanospheres, such as inhibiting the uptake of nanospheres through the reticuloendothelial system and increasing drug aggregation. Huang et al. reported an cRGD peptide-modified PEGylated nanocrystal (NC@PDA-PEG-RGD) that has the function of targeting lung cancer cells [84]. In the evaluation of antitumor experiments, cRGD-modified nanocrystals have been found to be superior to unmodified or free drugs due to their higher intratumoral dosage and smaller tumor volume. Wu et al. established an integrin-targeted DDS (RGD-PDA-PHBV-PTX) for the treatment of hepatocellular carcinoma [85]. In this study, cRGD-modified NPs were observed to have stronger fluorescent signals than peptide-free groups in both HepG2 cells and mouse tumor tissues, and they were mainly distributed at tumor sites, suggesting that the accumulation of cRGD-modified NPs at tumor sites was significantly higher than that of non-targeted NPs, which might be due to αvβ3/αvβ5 integrin receptor and ligand interaction mediated targeting.

The NGR peptide (Asn-Gly-Arg) is a specific peptide that has the ability to target aminopeptidase N (CD13), which is upregulated in the angiogenic tumor vasculature and many kinds of tumor cells (e.g., human fibrosarcoma cells HT-1080), with an expression level approximately 10 times higher than that of healthy cells [86,87]. CD13 is a multifunctional protein associated with tumor angiogenesis [88]. Carriers conjugated with NGR peptides have successfully delivered cytotoxic drugs such as DOX, apoptotic peptides, and cytokines to tumors or tumor vasculatures, enhancing the antitumor activity [75,89,90,91]. Wang et al. developed a peptide-containing polymer micelle (NGR-PM-DTX) [91]. Spectral fluorescence and confocal image analysis showed that NGR promoted the uptake of micelles by CD13-overexpressing tumor cells (HT-1080). Further studies found that the NGR-PM-DTX group had higher cytotoxicity against HT-1080 cells, which was very consistent with the observation of cell uptake. In mice bearing HT-1080 tumors, the targeted nanoparticle group had a stronger antitumor effect and less weight change. Gu et al. prepared an NGR-modified acid-responsive liposome (DTX/NGR-PLL) [92]. In their cell uptake experiment, the uptake of C6/NGR-PLL by HT-1080 cells was much higher than that of C6/PLL, indicating that CD13 receptor mediation could greatly promote cellular uptake. In order to further evaluate the targeting ability of DTX/NGR-PLL in vivo, they used a fluorescent probe (NBD-PE probe) to label liposomes, and the green fluorescence of NBD/NGR-PLL in tumor cells was higher than that of NBD/PLL, indicating that NGR-modified liposomes have strong targeting ability. In addition, the tumor volume of the DTX/NGR-PLL group was significantly smaller than that of the DTX/PLL group, indicating that the DTX/NGR-PLL group had a more significant inhibitory effect on tumor growth. Chen et al. designed a tumor-targeting nanoparticle (LPD-PEG-NGR/siRNA) [93]. Compared to the LPD-PEG-ARA (control peptide), LPD-PEG-NGR-treated HT-1080 cells had a higher uptake of siRNA, suggesting that NGR ligands improved the delivery efficiency of NPs. There was almost no fluorescence signal in the tumor tissues of tumor-bearing mice treated with LPD-PEG-ARA/siRNA, while LPD-PEG-NGR/siRNA showed a strong fluorescent signal, and the growth of HT-1080 tumors was inhibited, indicating that LPD-PEG-NGR had good tumor targeting. Luo et al. reported an NGR-modified liposome (NGR-SSL-PTX) [94]; the results showed that NGR-modified liposomes had the ability to specifically target tumor endothelial cells and tumor cells, thus exhibiting the dual effects of inhibiting angiogenesis and inhibiting tumor growth.

The T7 peptide (HAIYPRH) is a transferrin receptor (TfR)-binding peptide that has a strong affinity for TfRs (the expression level on tumor cells is about seven times that of healthy cells), and this binding force is very similar to transferrin, but they have different binding sites [95,96,97]. Based on this, Lu et al. constructed a tumor-targeting cationic gene carrier (CRD-PEG-T7) for the targeted delivery of plasmid DNA to treat prostate cancer [98]. The accumulation of T7 peptide complexes primarily occurs within tumors as a result of the bone-targeting properties of the aspartate sequence, the tumor cell-specific targeting ability of the T7 peptide, and the EPR effect. Compared with non-targeted plasmid DNA complexes, CRD-PEG-T7 could target tumor cells at a 4-fold higher rate than that of unmodified. Kim et al. modified exosomes with T7 peptide and the rabies virus glycoprotein (RVG) peptide, respectively, and then loaded antisense miRNA oligonucleotide (Amo-21) into exosomes to obtain T7-exo and RVG-exo DDSs [99]. T7-exo possessed a greater affinity for the brain, and it substantially diminished miR-21 levels in glioblastoma when compared to unmodified exo and RVG-exo.

##### Cell-Penetrating Peptides

Cell-penetrating peptides (CPPs) are a class of polypeptides that can carry macromolecular substances (e.g., proteins, siRNAs, and nucleotides) into cells, containing 5 to 30 amino acids [100]. Depending on their physical properties, they can be divided into three categories: cationic peptides (e.g., TAT and penetrating proteins) [101,102], hydrophobic polypeptides (PFVYLI) [100], and amphiphilic polypeptides (e.g., Pep-1 and MAP) [103,104]. The transmembrane mechanism of CPPs is energy-dependent entosis and energy-dependent direct osmosis [105]. Their biggest advantage is that they do not damage the cell membrane.

**Figure 2:**
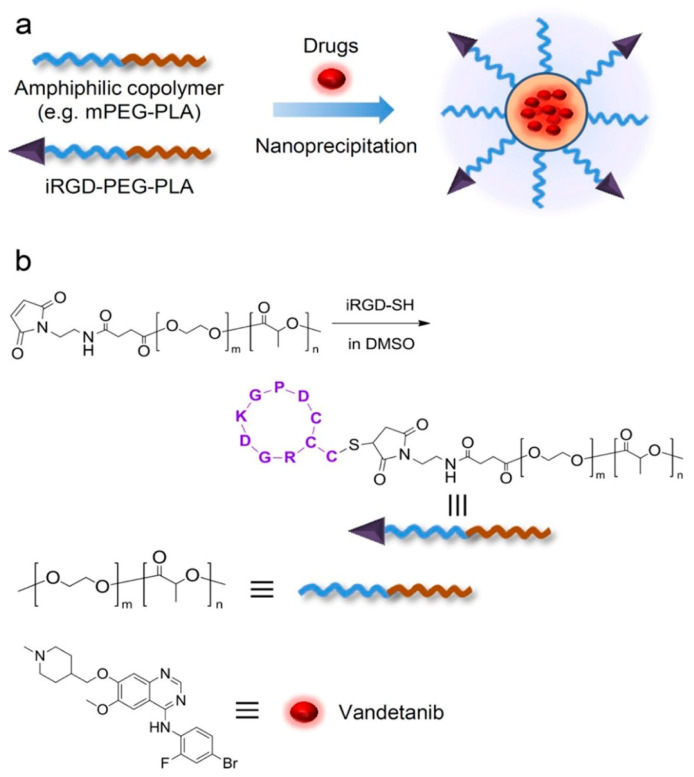
Development of systemically injectable NPs based on amphiphilic block copolymers for the tumor-specific in vivo delivery of molecularly targeted agents. (**a**) Schematic illustration showing the reformulation and self-assembly of vandetanib (vanib) into PEG–PLA NPs. To confer tumor-targeting functionality, a peptide ligand, iRGD, was incorporated into the surface of NPs in a co-assembly fashion. The iRGD-modified PEG–PLA was generated via thiol–maleimide coupling. (**b**) Molecular structures of iRGD–PEG-PLA, mPEG–PLA, and vanib. Adapted with permission from ref [113]. Copyright © 2016 American Chemical Society.

iRGD (CRGDKGPDC) is an RGD-derived peptide consisting of an RGD sequence that binds integrin αγβ3 and a recessive regular (CendR) sequence that binds extravasation and tissue osmosis associated with neuropilin-1 (NRP-1) [33,106], which is a non-tyrosine kinase receptor that exists in excess on the outer surface of glioma cells and angiogenic endothelial cells, with an expression level more than two times that of healthy cells [107,108]. Intravenous iRGD was first used to target integrins in the tumor vasculature [109], and then iRGD was proteolytically proteolyzed into CRGDK/R, exposing the active CendR sequence and the binding of this sequence to NRP-1, which triggers transport channels across the vascular wall and tumor tissue surface (Transcytosis) [110]. As a result, this system allows drugs that are chemically coupled with iRGD, or even physically entrapped, to permeate and spread inside tumor tissues, thereby improving the therapeutic effect of anticancer drugs to a certain extent [110,111]. Wang et al. reported a peptide-functionalized thermocarbonized porous silicon (TCPSi) nanoparticle for targeted drug delivery to the tumor neovascularization [112]. The iRGD peptide was conjugated to bicyclic acyl-functionalized PSi NPs via a SPAAC reaction with a conjugation efficiency of 3.4% (molar ratio). It was observed from cell uptake and distribution experiments that the fluorescence intensity of peptide-containing NPs (ca. 62% of the treated cells associated with the NPs) was stronger than that of peptide-free NPs (ca. 52%), indicating that the peptide modification improved the uptake efficiency of TCPSi NPs in cells, thereby enhancing the antiproliferative effect in vitro. Wang et al. designed and constructed an iRGD peptide-modified systemic injectable nanoparticle based on amphiphilic block copolymers (PEG-PLA) for targeting hepatoma cells (Figure 2) [113]. According to the expression of CD31 in tumor tissues, targeted nanopreparations had lower vascular density than non-targeted nanopreparations. The targeted formulation significantly reduced the proportion of K_i_-67 positive cells in tumor tissue (15 ± 6.1%) compared to the non-targeted formulation (48 ± 8.3%). At the same dose (20 mg/kg), the apoptosis rate of non-targeted nanopreparations was only 18.33 ± 3.33%, while that of targeted nanopreparations was as high as 39.37 ± 5.89%. In conclusion, in vivo experiments have shown that targeted NPs can reduce microvascular density, increase cell proliferation inhibition, and promote apoptosis, reflecting their more effective tumor suppressive effects.

The density of peptide ligands on the nanocarrier is the main factor affecting the functions of peptide-conjugated carriers such as transcytosis, adhesion, and cellular response. Yin et al. synthesized three cylindrical polymer brushes (CPBs) with different iRGD coupling densities, in which the contents of iRGD were 0% (Peptide-free), 50%, and 100%, respectively [114]. Based on the results of cell uptake, biodistribution, and tumor spheroid permeability experiments, it was found that the transcytosis of NPs gradually enhanced with increasing density of the peptide ligands, resulting in deeper tumor spheroid penetration (100 μm) and more drug accumulation. Liu et al. demonstrated that increasing the density of RGD domains in crosslinked membranes led to more robust cell adhesion and diffusion [115]. The same polyethylene terephthalate (PET) material was grafted with different densities of RGDC peptides: when the RGDC density exceeded 1 ± 0.2 pmol/mm^2^, osteoblasts and endothelial cells showed responses [116]. Therefore, precise quantification and regulation of the peptide density are crucial to ensure the preparation of peptide–DDSs with the functional properties required for anticancer drug delivery. These research projects have revealed that increasing the ligand density could enhance the rate of transcytosis.

iNGR (CRNGRGPDC) is another CPP, which is similar to iRGD peptides, except that their tumor-targeting motifs are different. In order to increase the penetration of antitumor drugs into glioma parenchyma, Kang et al. chemically combined iNGR with PEGylated PLGA NPs [67]. The peptide coupling efficiency determined by the BCA protein assay was 60.3 ± 4.7%, and the peptide density on the surface of the NPs was calculated to be 627 ± 58 S. In glioma distribution and penetration experiments, iNGR-NPs had the highest accumulation and deepest penetration at the tumor site, accounting for about 25% of the total fluorescence signals detected in the range of 0–10 µm, and about 46% of the fluorescence signals detected in the range of more than 20 µm of penetration, while the total fluorescence intensity of peptide-free NPs was only 35.6 ± 4.9% of the total iNGR-NPs, and more than 50% were captured in the range of 0~10 mm from the blood vessels. In intracranial glioma mice, the antiangiogenic activity of iNGR-NPs loaded with PTX was improved, resulting in significantly prolonged survival time of the mice. Zhou et al. prepared iNGR-modified liposomes (iNGR-SSL) [117]. The results showed that the accumulation of iNGR-SSL/DiR in tumor tissues was significantly increased compared with the unmodified liposomes, indicating that iNGR peptides have good targeting ability. In addition, in the glioblastoma model, iNGR-SSL/DiR showed a significantly longer and increased distribution compared to NGR-SSL/DiR (43 days), and this ultimately led to the strongest antitumor effect and the longest survival time (50 days) in this group of mice, which should be attributed to the penetrating effect of iNGR in tumor tissue.

**Figure 3:**
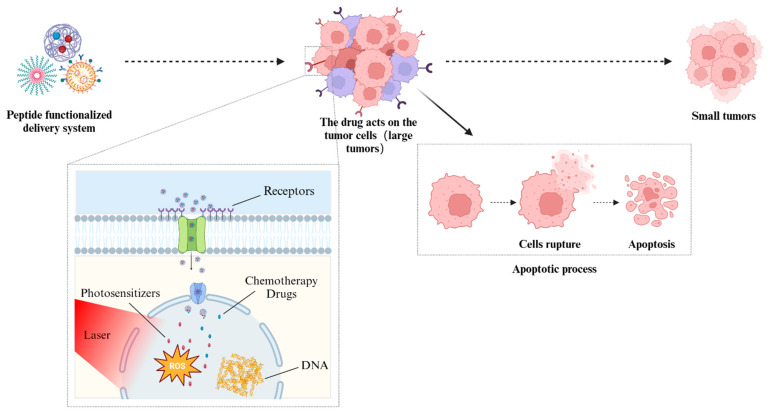
Peptide functionalized delivery systems (containing photosensitizers and chemotherapeutic agents) act on tumors. The peptide functionalized delivery system targets the nucleus of tumor cells, and the photosensitizer released produces reactive oxygen species under laser irradiation, which synergizes with chemotherapy drugs to kill tumor cells. The figure was prepared using BioRender, and a publication license was obtained.

tLyp-1 peptide (CGNKRTR), a ligand of the NRP receptor, contains tumor homing motifs and CendR motifs [118,119]. There have been many studies done to introduce it into nanoformulations to improve antitumor efficiency. For example, Hu et al. conjugated tLyp-1 peptide to PEG-PLA (tLyp-1-NP) to endow them with tumor homing and vascular extravasation [118]. XPS analysis confirmed that the nitrogen content (0.53%) detected on the surface of tLyp-1-NP was contributed by peptides. The modified NPs showed enhanced cellular uptake and increased drug cytotoxicity in HUVEC cells and rat glioma cells. More importantly, significant improvements in tumor selective accumulation and penetration of tLyp-1-NP (1.32 times greater than that of unmodified NPs) were observed in both C6 glioma spheres without blood vessels and in intracranial C6 glioma mice. Due to the enhanced antitumor efficacy of peptide-modified NPs, tLyp-1-NP-PTX treatment obtained the longest survival (the medium survival time was 37 days), while NP-PTX was only 28 days. Jiang and colleagues modified polylactic acid succinate NPs containing photosensitizers and chemotherapy drugs through the tlyp-1 peptide (tLyp-1 conjugation efficiency around 57%) to obtain a delivery system combined with photodynamic therapy (Figure 3) [120]. After laser irradiation, the peptide NPs exhibited high cell uptake and strong cytotoxicity against both HUVEC cells and DOX-resistant MCF-7/ADR cells. It is worth noting that the apoptosis rate caused by the tLyp-1-NP (18.59 ± 1.42%) was still higher than that of the NP (8.65 ± 1.73%) in the absence of laser. After intravenous administration, tLyp-1-NPs were found to present a more effective reversal of DOX resistance, as well as better tumor targeting and penetration ability, compared to unmodified NPs. Lei et al. used tLyP-1 modified tungsten disulfide quantum dots (WS_2_-HPs) and DOX-loaded mesoporous silica NPs (MSNs) to construct a nanomedicine (DOX@MSN-WS_2_-HP) with acid-responsive and size-variable properties [121]. It was noteworthy that the nanomedicine could rapidly decompose into positively charged DOX@MSN-NH_2_ and small-sized tLyP-1 peptide-modified WS_2_-HPs after accumulation within tumors. Unlike traditional tumor penetration strategies, the WS_2_-HPs used a hybrid strategy of fusion size control and functional peptide modification to enhance the penetration ability of tumor tissues. The experimental results showed that in the 4T1 tumor spheroids, the WS_2_-HP was observed to have a maximum tumor penetration depth of 94 um, while the peptide-free NPs were only about 61 μm. In ex vivo tumor tissues, unmodified NPs were mainly distributed in marginal regions, while the WS_2_-HP exhibited deep and uniform penetration in solid tumors.

RLW (RLWMRWYSPRTRAYG) is a CPP developed using mRNA display technology, which has strong cell penetration and high cell selectivity, as well as can selectively target A549 cells [70]. Gao et al. anchored RLW to PEG-poly(ε-caprolactone) (PCL) NPs for the treatment of lung cancer [70]. To study the uptake of RLW-NPs by cells, the researchers incubated them with A549 cells and HUVEC cells. The results showed that the fluorescence intensity of RLW NPs in A549 cells was higher than that in HUVEC cells (no significant difference with NPs), and the uptake capacity of A549 cells was 2.1 times that of NPs, indicating that RLW-NPs have significant cell selectivity and strong permeability. Further tumor penetration experiments showed that RLW-NPs were distributed in the core of the spheroids at the bottom 140 mm, while the fluorescence of NPs was only distributed in the periphery of the A549 spheroids, indicating that RLW binding could specifically improve the penetration of the A549 spheroids. Quantitative results also support this conclusion. Slice results showed that at 140mm away from the bottom of the A549 sphere, the distribution of RLW-NPs in the core farther from the surface was 17.04 times that of NPs, which further verified that RLW can specifically improve the penetration of the A549 sphere. In their anticell proliferation assay, the apoptosis rate of A549 cells treated with DTX-RLW-NPs was 4.5 times higher than that of the NPs group, suggesting that the specific targeting of A549 cells by RLW could improve apoptosis. However, this experiment demonstrated that RLW-modified NPs significantly enhanced DTX delivery to A549 lung cancer while reducing toxicity in vivo. However, it is important to note that tumors still grow rapidly (more than 1000 mm^3^ in 14 days), possibly due to the relatively large particle size of the NPs and the slow release of the drug. In another similar experiment, Yang et al. prepared a peptide-functionalized polymer with a surface peptide density of 18.7% [69]. Compared to the peptide-free panel, it not only guaranteed high permeability, but it also had the longest survival (75 days) and apoptosis of a large number of tumor cells.

F3 peptide (CKDEPQRRSARLSAKPAPPKPEPKPKKAPAKK) is a molecule that is efficiently transported from the tumor cell surface to the nucleus by binding to nucleolins [68]. Nucleolin is a shuttle protein found in many tumor cells and tumor-associated blood vessels that is responsible for transport between the cell membrane and the nucleus, providing an attractive target for mediating specific targeting and improving tumor permeability [71]. Wan et al. synthesized a new peptide CF by conjugating a CREKA molecule (tumor homing peptide) to F3 using an acid-reactive linker hydrazone, which they decorated on the surface of erlotinib (EB) and γ secretase inhibitor (DAPT) double-loaded NPs, and they successfully obtained functionalized NPs (CF-NP-EB/DARTs) with a nitrogen content of 0.56% [122]. Under normal physical conditions, F3 temporarily loses its ability to penetrate cells, making the cytotoxicity of the drug to normal tissues greatly reduced. When it enters the TME, acidic conditions break the bond between the CREKA and F3, thereby restoring their ability to penetrate cells. Mice with triple-negative breast cancer treated with CF-NP-EB/DAPT showed the most significant tumor accumulation, the strongest antitumor activity, and the smallest tumor volume compared to controls, including CREKA-NP-EB/DAPT. In another example, Hu et al. developed a nanoparticle functionalized with an F3 peptide (peptide coupling efficiency of 48.5 ± 2.3%) [68]. The experimental results showed that the F3-NP was more widely distributed than the NP without F3 in 3D tumor spheroids, and the penetration depth was deeper (139.26 mm), while the penetration depth of the NP was only 81.02 mm. In addition, a low distribution of the NP was observed at the site of glioma in the mice studied, mainly at the tumor margin. In contrast, the F3-NP achieved a significantly higher distribution and deeper penetration.

##### Nuclear Localization Signal Peptides

The nucleus, being the central hub of cellular metabolism and heredity, plays a crucial function in the processes of cell proliferation, metabolic activities, and cellular differentiation. The nucleus was selected as the ideal site for the primary role of most antineoplastic drugs and gene therapies [123]. Nuclear localization signaling (NLS) peptides are a class of small molecular peptides derived from natural proteins, which have been widely used to promote the entry of vectors into the nucleus [73,124].

Maity and Stepensky obtained core-shell CdSe-ZnS quantum dots (QDs) modified with an NLS peptide (PKKKRKVKAA) through three-stage modification, and the peptide coupling efficiency was 37.9% [125]. Intracellular targeting experiments showed that the accumulation in the nucleus or adjacent region of HeLa cells increased by 56.1% after NLS modification on the surfaces of the quantum dots. The preferential accumulation of QD-NLS suggested that NLS peptides could effectively target the nucleus as a targeting residue.

Based on the DNA replication mechanism, when DNA replication encounters repeated DNA sequences or abnormal sequences, the DNA double helix structure will break or fail to copy opposite DNA strands, resulting in the blockage of the DNA replication program and ultimately inhibiting cell proliferation. Wu et al. successfully prepared pH/enzyme double-sensitive dendritic polymer NPs, whose shells were composed of an NLS peptide (PKKKRKV), an enzyme-sensitive tetrapeptide (GFLG) and a pH response molecule morpholine (Mp) [126]. In nuclear localization studies, the red fluorescence signal in the nuclei of peptide (NLS)-modified NPs was enhanced for a short time, indicating that the nuclear targeting efficiency was higher than that of peptide-free NPs. After the treatment of mice, the tumor inhibition rate of the NLS-modified nanoparticle group reached 88.47%. Yu et al. successfully prepared polymer micelles (NCHGCs) by coupling an NLS peptide (Ac-CGYGPKKKRKVGG) with a cholesterol-modified glycol chitosan, and they used them to deliver DOX [127]. The degree of substitution of the NCHGC complex was 3.8 NLS per 100 sugar residues. DNCHGC micelles had higher drug loading and lower IC_50_ values (0.461 ug/mL) compared to non-NLS-modified micelles, as well as exhibited greater cellular uptake in HeLa nuclei.

TAT peptides (YGRKKRRQRRR) have been shown to be another effective molecule that can transport NPs into the nucleus by binding to the input protein α and β (nuclear mucin) [73]. Pan et al. coupled TAT peptide with mesoporous silica for nuclear targeted drug delivery [73]. Cytotoxicity and anticancer experiments confirmed that TAT conjugates with small diameters (less than 50 nm) can effectively transport active anticancer drugs into the nucleus and release drugs in a sustained and slow manner, resulting in a decline in cancer cell viability for a short period of time. Sethuraman and Bae developed an ultra-pH-sensitive polymeric targeting system for the treatment of acidic solid tumors [74]. Their nanomaterial PLLA-b-PEG was conjugated with TAT to form the active target nanocarrier. After achieving the tumor site, TAT was exposed to interact with tumor cells, and then the TAT micelles ended up on the surface of the nucleus.

##### Mitochondria-Targeting Peptides

Mitochondria form the structure in cells that make energy, as well as the main sites for aerobic respiration in cells. Besides, mitochondria are also involved in several processes such as cell differentiation, cell messaging, and apoptosis [128,129]. At present, mitochondria as a target for tumor therapy has become an emerging field. Some peptides, known as mitochondria-targeting peptides (MTPs), can disrupt the membranes of mitochondria and induce the apoptosis of tumor cells [130]. For example, (KlaKlaK)_2_, a common apoptotic peptide, is known as a membrane disruptor. Based on its subcellular organelle targeting ability, (KlaKlaK)_2_ has often been used in combination with cell penetrating parts or receptor ligands [75,131,132,133,134]. In the case of promoted uptake, the (KlaKlaK)_2_ peptide disrupted the integrity of the mitochondrial membrane and plasma membrane and initiated the apoptosis program [75,131,132,135].

Wang’s group reported a type of dual-response nanoreactors (DRONEs) grafted with tumor homing proapoptotic peptides (CGKRK_d_ [KlaKlaK]_2_) (THP) [133]. By using the HPLC and Bradford assays, the average percentage of peptide conjugation was calculated to be 28.47%. In these nanoreactors, the CGKRK sequence could bind to a large number of p32 proteins (the expression was about five times that of healthy cells) in glioblastoma cells [136,137]. Subsequently, the [KlaKlaK]_2_ sequence provided a targeting property, resulting in DRONEs to preferentially accumulate in mitochondria. Then, the photosensitizer was released to destroy the mitochondria and initiate the apoptotic signal. BBB penetration, biodistribution, and mouse survival experiments confirmed that DRONEs showed the advantages of excellent targeting in the treatment of glioblastoma. Law et al. synthesized a potent cytotoxic peptide r7-(KlaKlaK)_2_ by binding (KlaKlaK)_2_ to the cell-penetrating domain r7 [134]. In just a few minutes, the morphological changes of the cells and the leakage of material within the mitochondria were evident under the microscope and were consistent with rapid cell apoptosis. Standley developed a novel amphiphilic molecule (PA) containing the (KlaKlaK)_2_ peptide [138], which could self-assemble into nanofibers with the peptide sequences dispersing on the surface. In cell experiments, the reduction in the plasma membrane and mitochondrial membrane potential suggested that (KlaKlaK)_2_ PA could effectively disrupt membrane integrity to activate the cell death pathway.

#### 2.1.2. Stimulus-Responsive Peptides

Due to the high expression of certain components within tumors, the responsiveness of peptides had been widely used to construct stimulus-responsive DDSs. When these responsive systems reached tumor tissue, they were stimulated by the abnormal microenvironment within the tissue (such as high GSH levels, certain over-expressed enzymes, low oxygen levels, and low pH) to release the drug. Therefore, it was particularly important to construct efficient stimulus-responsive peptide-based nanocarriers that could respond to environmental parameters for drug delivery. Table 3 lists some of the stimulus-responsive peptide-based nanomaterials that have been reported for tumor therapy.

##### pH-Responsive

Tumor tissues are in a situation of high lactate content due to high nutrition metabolites, and the pH value of the tumor tissue is lower than that of normal tissues or organs (7.4) [153]. For drug delivery, acid-responsive drug carriers are designed, which help the vector to respond to H^+^ at the tumor site, avoiding the premature degradation of the drug in the circulation process and enhancing drug release [154].

Two main strategies for designing pH-responsive components have been identified: In the first method, an acid-responsive peptide is often coupled to the backbone of other materials, triggering the protonation of the polypeptide under acidic conditions and resulting in a positive charge on the surfaces of NPs. This change promotes the rupture of the endosomal membrane and enhances the release of the drug from the endosomal to the cytoplasm [155]. In the second method, the peptides typically act as the backbone whose secondary structure responds to low pH, causing all or part of the carrier to undergo conformational changes that undermine the integrity of the nanostructure [156].

For the first strategy, commonly used cationic peptides include poly(lysine) (PLL) and poly(histidine), because they have amine groups that can be ionized. Lee et al. reported a gene delivery system PAsp (DET-Aco) [157]. The system was negatively charged at extracellular pH but turned positive at endosomal acidic pH to disrupt the endosomal membrane. The coating of PAsp (DET-Aco) onto positively charged PLL and poly(l-ornithine) mRNA-polyplex significantly improved the endosomal escape and thereby improved the protein expression [158]. Yang et al. synthesized a pH-responsive DDS by modifying mesoporous silica NPs (MS NPs) with L-PLL and polyglutamic acid (L-PGA), respectively, in which peptides acted as “switches” to control drug release [159]. The modified PLL-MS NPs and PGA-MS NPs self-assembled into stable particles in a neutral environment. When the pH was reduced, the L-PLL became unstable and underwent protonation, promoting the fusion of NPs with the membrane and the release of the drug. It is worth noting that the rate at which the drug was released was not the same at different levels of acidity. In the methylene Blue (MB) model drug release experiment, when the pH value decreased from 7.4 to 4.5, the MB release rates of MB@PLL-MS NPs and MB@PGA-MS NPs, as well as mixed groups, increased with the decrease in pH value, among which the mixed group had the highest release rates (37–56%). When the ratio of PLL-MS NPs to PGA-MS NPs was 1:1, 88% of the HeLa cells were killed by DOX.

For the second strategy, the secondary structure of the peptide underwent a transformation with a change in the pH, which was related to the hydrophilic block in the copolymer structure. With the increase in the hydrophilic weight fraction, the interface curvature of the solution morphology increased [160]. Savin’s group reported three triblock copolymers with high hydrophilic weight fractions (greater than 75%), namely, KPK27, KPK46, and KPK52 [161]. Due to the change in pH (3–7), a significant morphological shift occurred in each case. The triblock of KPK 27 and KPK 52 underwent a transition from spherical micelles to vesicle, while KPK 46 went from a spherical micelle to a discoid micelle. The morphology transformation could be explained by the critical phase behavior. It is well known that the self-assembly behavior could be affect by the hydrophilic/hydrophobic volume fraction (ρ) of nanocarriers. Upon increasing the hydrophobic volume fraction, the morphology of aggregates could change to a disordered structure, spheres, cylinders, gyroid, and lamellae. For polypeptides KPK27, KPK52, and KPK46 with high-lysine weight fractions, they exhibited morphology transitions as a pH responsibility. Upon increasing the pH value, the polypeptides were deprotonated, the surface charge was changed from positive to neutral, the hydrophobic fraction was increased, and the interfacial curvature was decreased; therefore, the morphology of the aggregates was transferred from spheres to vesicle or disk. Amphiphilic copolypeptides α-helical PLeu-*co*-PLL formed vesicles under basic conditions; upon decreasing the pH value, the protonation of PLL residues induced a change in both the hydrophobicity and secondary structure, resulting in the release of cargoes [162]. Han et al. reported an acid-responsive chimeric peptide with the peptide sequence AEAEAKAKAEAEAKAK [142]. Under physiological conditions, chimeric peptides were self-assembled into spherical NPs. In the TME, the 2,3-dimethylmaleic anhydride group for charge shielding was removed in response to acidity, followed by ionic complementation between peptides to form rod-shaped NPs. This acid-triggered geometric transformation gave chimeric peptides that accelerated uptake by tumor cells.

Yamamoto et al. synthesized a novel amphiphilic peptide C_16_-VVAEEE (C_16_ stands for hexadecanic acid) that could be self-assembled into entangled nanofibers from micelles under intracellular pH values [143]. The cytotoxicity of the hydrogel was controlled by acidity, such as at low pH values (6.8–7.1), it could cause a large number of cancer cells to die, and the cell activity was reduced by about 70% to 80%.

In addition to the above-mentioned ones, there are other PH-responsive peptide-based delivery systems. For example, Raza and colleagues developed a pH-sensitive FER-8 peptide (FEFEFRFK) hydrogel delivery system (HG-PTX) with a peptide purity of up to 97.34% [144]. At an acidic pH, the hydrogel structure was degraded, and PTX was released. Compared with free PTX, the HG-PTX increased drug accumulation within HepG2 cells, inhibited cell growth, and prolonged residence time (96 h).

##### Enzyme-Responsive

Enzymes are catalytic substances being generated by living cells. Almost all metabolic reactions in the body depended on enzymes, and the control of material metabolism was also achieved through the regulation of the enzyme activity [163]. It was further found that there was an overexpression of certain enzymes in the tumor [164], such as matrix metalloproteinases (MMPs) [146,165], caspase [166], lysyl oxidase (LO) [152], and cysteine cathepsin [147,148,149]. Therefore, these representative high-expression enzymes are excellent candidates for designing enzyme-responsive drug vectors.

MMPs, a group of proteases abundant around tumors, have been related to tumor angiogenesis and tumor cell metastasis [167]. At total of 26 members of the MMPs family were isolated and identified, among which MMP-2, MMP-7, and MMP-9 were found in tumors, and their contents were much higher than those of normal cells [168,169]. The GPLGIAG sequence could be cut by MMPs in the TME, enhancing the cellular uptake rate of the NPs. Guo et al. reported an MMP-2-responsive nanoparticle (mPEG-GPLGIAGQr9-PCL/Cur) for improving the in vivo bioavailability of curcumin, and the peptide GPLGIAGQ was utilized as the enzymatic responsive sequence [170]. When the NPs reached the tumor, GPLGIAGQ underwent structural degradation in response to MMP-2, exposing the NPs to interact with the cell. In vitro drug release experiments also further verified that the internal diffusion channels formed by enzyme shear reactions contribute to accelerating drug release behavior [145]. Moreover, A549 nude mice treated with these NPs showed the strongest tumor inhibition effect (76.95%). Cao’s group synthesized a surfactant-like peptide (Nap-FFGPLGLARKRK), in which GPLGLA was an enzyme-sensitive fragment of MMP-7 [146]. Peptides self-assembled to form long fibrils to load DOX. When these drug-loaded fibrils reached the tumor site and contacted with overexpressed MMP-7, the fibrils changed from thicker fibrils to thinner fibrils to release DOX. In vivo antitumor experiments in mice demonstrated the high efficiency of this enzymatically reactive peptide carrier in successfully inhibiting tumor cell proliferation and differentiation while greatly reducing side effects. Wu et al. prepared an enzyme-reactive invisible peptide coating gold nanorods (peptide content of 7%), and GPLG was utilized as the MMP-9 response peptide [165]. When the modified gold nanorods were circulated to the tumor, MMP-9 caused GPLG breaks and detachments in the nanorod structure. In the presence of MMP-9, responsive gold nanorods showed higher cellular uptake in vitro and more accumulation within tumors.

Cathepsin B (CB) is a natural protease that is ubiquitous within cells, especially in tumor cells. As early as 1984, Kopecek had found that the four-sequence polypeptide GFLG could be hydrolyzed by CB [171]. Jin et al. synthesized a peptide (CC-EKEK-V-FrFKFrFK-V-GFLG-V-EKEKEKEKEKEKEKEKEKEK) in response to cathepsin B and used as a ligand to modify gold nanorods (AUNR) [147]. The CB response sequence GFLG served as a linker to connect the hydrophobic and hydrophilic chains. When the peptide gold nanorods entered tumor cells, the GFLG sequence was cleaved by CB, gold nanorods target mitochondria, and accumulate. Under 808 nm near-infrared laser irradiation, the apoptosis caused by responsive peptide gold nanorods was significantly more than that caused by non-responsiveness. Zeng et al. introduced enzyme response sequences to construct a peptide prodrug (HCPT-FF-GFLG-EEYSA) that could undergo morphological transformation for bladder tumor-specific administration [148]. After the peptide prodrug was internalized into the cytoplasm, its structure was destroyed by CB and finally released the drug completely.

Enterokinase is also a protease over-expressed on the mitochondria of tumor cells that causes a morphological transition of negatively charged peptide assemblies from micelles to nanofibers [172,173]. He and colleagues developed an enzymatically reactive mitochondria-targeting peptide for encapsulating and protecting magnetic NPs (MNPs) [150]. While peptides molecules spontaneously form amorphous nanoaggregates in an aqueous solution, MNPs are enclosed in hydrophobic centers by non-covalent interactions. When aggregates selectively accumulate on the mitochondrial surface and come into contact with enterokinase, the peptide assembly morphology changes, i.e., from nanofibers to nanofilaments, forming a peptide network that retains MNPs. Once MNPs are released, they can activate mitochondria to produce ROS, which leads to mitochondrial dysfunction and promotes apoptosis.

## 3. The Effect of Polypeptides-Based Nanocarriers on Immune Cells

It has been reported that the occurrence and development of tumors are closely related to the body’s immune function [174]. According to the tumor immunology editing hypothesis, the relationship between the two can be divided into three stages: immune clearance, immune equilibrium, and immune evasion [175]. Tumor immunoediting is a dynamic process that explains the dual role of the immune system in body protection and tumor shaping, and it lays the foundation for individualized cancer immunotherapy. Tumor immunotherapy is a type of treatment that fights tumors by activating or reshaping the body’s immune system. However, its safety and efficacy still face several challenges, including inefficient immune responses, serious toxic side effects, and limited retention time of immune adjuvants in the TME. The combination of nanoformulation and immunoadjuvant delivery has become a trend due to its improved efficacy and alleviation of adverse effects in non-targeted organs and tissues.

### 3.1. Peptide-Functionalized Drug Delivery Carriers

**Figure 4:**
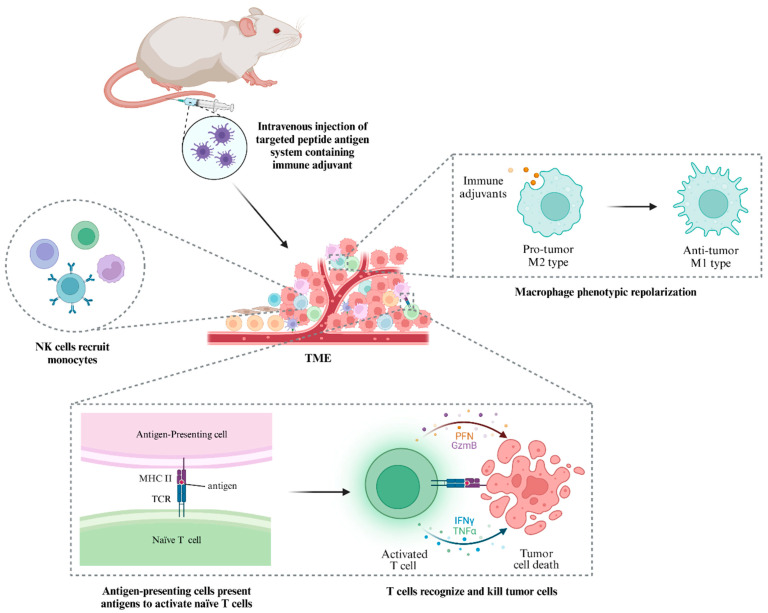
Tumor immune response induced by peptide-functionalized delivery system. Targeted peptide–antigen system containing immune adjuvants was injected intravenously into the cancer mice. It reaches the TME through the blood circulation and decomposes. The antigenic peptides are taken up by APCs and presented to the initial T cells. Activated T cells begin to recognize tumor antigens and release cytokines. The released immune adjuvant is phagocytosed by macrophages and phenotypic repolarization to antitumor M1 TAMs (M1-like TAMs). At the same time, activated NK cells began to recruit monocytes and cooperate with T cells and macrophages to attack tumor cells. The figure was prepared using BioRender, and publication license was obtained.

In addition to playing effects on tumor cells, peptides also have an effect on immune cells. T cells, NK cells, and TAMs are the primary immune cells [176]. These three systems interact and communicate with each other to inhibit the development of tumor cells, so they are crucial in tumor immunotherapy (Figure 4). The following summary will focus on the relationship between these three types of immune cells and tumors and the peptide functionalized delivery system. Table 4 lists peptide-based nanocomplexes for delivering immune molecules targeting different receptors/pathways.

#### 3.1.1. Effect on T Cells

T lymphocytes are referred to as T cells, and the initial T cells enter the blood circulation after maturing in the thymus, settle in the peripheral lymphoid organs, and recycle in the body to exert cellular immunity and immune regulation functions. T cells amplify and enhance the immune response by secreting and releasing a variety of cytokines, such as interferon, thus playing an irreplaceable role in stopping the formation and progression of tumors [176]. They can be divided into various subpopulations based on their different functions in the immune response. Among them, cytotoxic T lymphocytes (CTLs) are the main effector cells, which can efficiently and specifically kill tumor cells through two pathways: perforin/granzyme and death receptor.

In order to improve the efficiency of antigen uptake by APCs, Wu et al. successfully prepared Clip6-OVA by conjugating endogenous peptide 6 (Clip6: KVRVRVRV^D^PPTRVRERVK-NH_2_) with ovalbumin (OVA) using cytosol [180]. The fluorescent signal of mouse lymph nodes in the conjugate was more than five times that of OVA alone, revealing the better efficiency of antigen uptake by APCs. Moreover, when combined with an immune adjuvant (CpG), the crosspresentation of protein antigens was further enhanced, and finally, a stronger CTL-mediated immune response was obtained. Liu et al. reported a polylactic acid–hydroxyglycolic acid (PLGA) nanoparticle for encapsulation of OVA (MPG^ΔNLS^-OVA conjugate) modified by MPG^ΔNLS^, which is a type of CPP [181]. In vitro experiments showed that peptide-containing NPs enhanced antigen crosspresentation, thereby stimulating the expansion of ova-specific T cells, the production of ova-specific IgG antibodies, and the proliferation of ova-specific memory T cells. In addition, peptide-containing NPs treated with tumor-bearing mice could significantly inhibit tumor growth and prolong the survival of mice. Kuai et al. reported a high-density lipoprotein nanodisk (sHDL) consisting of phospholipids and apolipoprotein A1 mimics peptides [182]. The peptide nanodisks promoted APC maturation and the crossactivation of CD8α+ T cells in vitro. After administration, the frequency of neoantigen-specific CTL induced by peptide nanodisks (sHDL-Adpgk/CpG group) was 47 times higher than that of the soluble vaccine (Adpgk + CpG group) and 31 times higher than that of the strongest adjuvant (Adpgk + CpG + montanide group) after the administration of MC-38 to colon cancer mice. Trabbic et al. described gold NPs (AuNPs) based on the enzyme response peptide sequence (GFLG) for targeting APCs expressing Dectin-1 [183]. These particles could stimulate the production of high-titer antibodies to trigger an immune response; moreover, the released cytokines produced a good antitumor balance. Fries et al. developed an end-sealing peptide for controlling the length of spiral peptide nanofibers [190]. Compared with the nanofibers without end-blocking peptides, the nanofibers were preferentially crosspresented by dendritic cells. Due to enhanced crosspresentation, these nanofibers containing end-blocking peptides triggered a stronger CD8+ T cell response in mice than nanofibers without end-blocking peptides.

**Figure 5:**
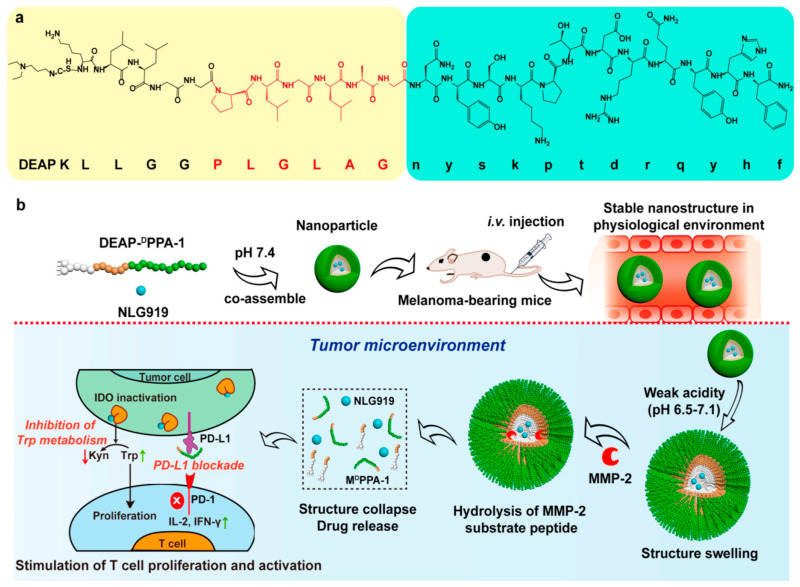
Composition of DEAP-^D^PPA-1 and the antitumor mechanism of NLG919@DEAP-^D^PPA-1 NPs. (**a**) The amino acid composition of DEAP-^D^PPA-1. The hydrophobic domain contains a functional DEAP molecule and an MMP-2 substrate with one lysine, two leucines, and two glycines as a linker. ^D^PPA-1 constitutes the hydrophilic domain. (**b**) The proposed antitumor mechanism of the NLG919@DEAP-^D^PPA-1 nanoparticle. DEAP-^D^PPA-1 and NLG919 co-assemble into a nanoparticle that maintains stable nanostructure at physiological environment. Upon penetration into the tumor stroma, the hydrophobic core of the nanoparticle swells in response to tumor acidity, allowing MMP-2 to access and hydrolyze its substrate peptide and leading to the complete dissociation of the nanostructure. Thereafter, NLG919 and M^D^PPA-1 are released for targeting IDO and PD-L1, respectively. Adapted with permission from ref [191]. Copyright © 2018 American Chemical Society.

Cheng et al. designed a therapeutic peptide-assembled nanoparticle that could accept dual stimuli sequentially and respond accordingly in the extracellular matrix of tumor cells, enabling the targeted delivery of ^D^PPA-1 (a short-peptide inhibitor of PD-L1) and NLG919 (an IDO inhibitor) to the tumor site for enhanced immunotherapy [191]. They first synthesized an amphiphilic peptide consisting of DEAP (3-diethylaminopropyl isothiocyanic acid), a substrate peptide of MMP-2 (matrix metalloproteinase-2), and ^D^PPA-1, and they then co-assembled it with NLG919 to form NPs through hydrophobic interactions. After intravenous injection, these NPs could effectively accumulate at the tumor site via the EPR effect. Then, in a weak acidic environment, the DEAP molecules underwent protonation, causing the nanostructure to swell and allowing the MMP-2, which was highly expressed in the tumor matrix, to enter and cut the peptide substrate, further breaking down the NPs and releasing the ^D^PPA-1 and NLG919 (Figure 5). In all experimental groups, NLG919@DEAP-^D^PPA-1 NPs showed the best inhibitory effect on tumor growth (tumor volume less than 500 mm^2^ within 14 days) and significantly increased the proportion of CD8+ T cells and cytotoxic T cells producing interferon-γ (IFN-γ) in the tumor tissue. Additionally, NLG919@DEAP-^D^PPA-1 nanoparticle therapy significantly prolonged the survival times of the mice. These results indicated that NLG919@DEAP-^D^PPA-1 NPs can induce a powerful antitumor immune response by enhancing the survival and activation of tumor-infiltrating T cells.

Lynn et al. developed a peptide-TLR-7/8a nanoparticle (SNP-7/8a) that could be self-assembled by chemical programming [192]. SNP-7/8a induced more lymph node APC accumulation and a higher intensity CD8+ T cell response compared to peptide-free NPs. In addition, only SNP-7/8a elicited CD4+ T cell responses. Animals treated with the SNP-7/8a vaccine, which includes the neoantigen M07 or M21, showed enhanced tumor growth control compared to the control group.

#### 3.1.2. Effect on TAMs

**Figure 6:**
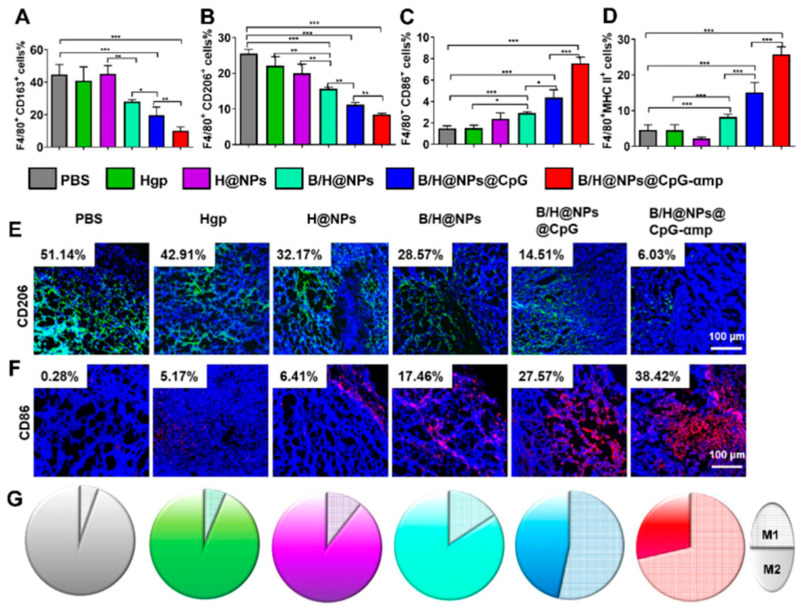
The TAMs phenotype reversion at tumor sites after treatment by different nanocomplexes. The M2-like TAMs surface markers CD163 (**A**) and CD206 markers (**B**), together with M1-like surface markers CD86 (**C**) and MHC II markers (**D**) of TAMs, were analyzed using flow cytometry. (**E**,**F**) The TAMs of M2-like (CD206) and M1-like (CD86) phenotypes were detected by immunofluorescence. Blue: cell nucleus; red: CD86 cells; green: CD206 cells. (**G**) The TAM phenotypes of M1-like (single-color fill) and M2-like (hatched grid fill) area fractions within tumor tissue as represented, respectively, by CD86 and CD206 biomarkers after treatment with different nanocomplexes. The data were analyzed using automatic multispectral imaging system (PerkinElmer Vectra II). Scale bar: 100 um. Three mice were analyzed in every group (n = 3), and one representative image is displayed per group. Data are expressed as the mean ± standard error of the mean (SEM). Differences between two groups were tested using an unpaired, two-tailed Student’s *t*-test. Differences among multiple groups were tested with one-way ANOVA followed by Tukey’s multiple comparison. Significant differences between groups are expressed as follows: * *p* < 0.05, ** *p* < 0.01, or *** *p* < 0.001. Reproduced from Reference [195] with permission. Copyright © 2022.

TAMs refer to macrophages infiltrating tumor tissues, which are differentiated and developed from monocytes and play a very important role in the host immune defense, surveillance, and self-stability; they are mainly divided into two phenotypes: antitumor M1-like and protumor M2-like. TAMs have both promoting and inhibitory effects on tumor immunity. In terms of the promoting effect, the M1-like can synthesize and secrete interleukin-12 (IL-12), induce NK cell activation, and significantly enhance its antitumor effect. From the point of view of direct action, the M2-like secretes a variety of immunosuppressive factors to downregulate the expression of co-stimulatory molecules and inhibit the activation of immune cells, thus promoting the development of tumors and treatment resistance [193]; In terms of indirect effects, the immune activity of T cells is generally indirectly inhibited by affecting other immune cells (e.g., Tregs, DCs, or MDSCs) [194]. In summary, in the TME, a variety of factors promote the increase in the number of M2-like TAMs, block specific immune responses, and accelerate tumor development. Therefore, the development of TAMs-targeted drugs can help reverse the immunosuppressive microenvironment and improve antitumor efficacy. Conde et al. reported a SiRNA-encapsulated peptide-functionalized gold nanoparticle (RNAi-M2pep-AuNPs) [185]. After 14 days of drug treatment, the fluorescence intensity of peptide-functionalized gold NPs in the macrophages of mice with lung cancer was stronger (90%) than that of the non-peptide group, and VEGF expression was reduced by 80%, indicating that peptide-functionalized gold NPs could specifically target TAMs and effectively silence VEGF mRNA. At day 21, the number of M2-like TAMs in the peptide-containing group decreased by 95%. In addition, the incidence of tumor cell cloning was significantly reduced, and the survival rate of mice (~75%) was increased. Qian et al. developed a nanoparticle (M2NPs-siCD115) with dual targeting of M2-like TAMs, whose structure and function were controlled jointly by M2pep (YEQDPWGVKWWY) and an α peptide (FAEKFKEAVKDYFAKFWD) [186]. After intravenous administration, M2NPs-siCD115 effectively targeted M2-like TAMs and blocked the CSFs-1/CSFs-1R pathway of the M2-like-TAMs, significantly eliminating the M2 TAMs in tumors (52%), thereby restoring CD8+ T cell infiltration (about 2.9-fold) and inhibiting melanoma cell growth (87%). In another paper, Han et al. also selected Mpep and α peptides to modify a polydopamine composite nanoparticle containing baicalin, an antigen Hgp peptide fragment, and a CpG fragment, and they successfully obtained a macrophage dual-targeted drug delivery system (B/H@NPs@CpG-αMp) [195]. Compared with nanocomposites lacking targeted ligands, dual-targeted nanocomposites had significantly stronger fluorescence intensities in M2-like TAMs and higher affinities for M2 TAMs. Furthermore, the uptake rate of dual-targeted NPs in M2-like TAMs were higher than that of M1-like. In vivo targeting results showed that M2-like TAMs captured three times more dual-targeted nanocomposites than non-targeted NPs. Due to the excellent dual-targeting ability of the nanocomplex, the drug was rapidly and effectively delivered to the tumor site, successfully reversing the macrophage phenotype (Figure 6) and eliciting an antitumor immune response and significant tumor growth inhibition (73.5%).

He et al. prepared a fusion peptide-functionalized gene delivery system (PHNP) targeting macrophages [196]. The PHNP system could effectively immunomodulate J774A.1 cells, repolarizing the M2 phenotype to an antitumor M1 phenotype while enhancing the secretion of proinflammatory cytokines and increasing the expression of M1 markers. Compared to NPs without peptide modification, PHNPs were delivered more efficiently, and the mediated transfection could be more effective in upregulating IL-12 and downregulating IL-10 and IL-4.

In a recent interesting report, Chen et al. constructed a chimeric peptide (KYEQDPWGVKWWYK) drug loading system (chip/RS) that encapsulated the resiquimod and SHP-2 inhibitor (SHP099) [187]. The interaction between CD47 on tumor cell surfaces and the signal-regulatory protein α (Sirp α) on TAMs triggered the activation of Src homology 2 (SH2) domain-containing tyrosine phosphatase 2 (SHP-2), initiating a “don’t eat me” signal to evade macrophage phagocytosis [197,198,199]. So, resiquimod was used in this study to repolarize TAMs to reverse the immunosuppressive microenvironment. Subsequently, SHP099 downregulated SHP-2 to promote the phagocytosis and clearance of tumor by M1-like TAMs.

#### 3.1.3. Effect on NK Cells

NK cells are immune cells with powerful antitumor functions in the body, and they are most similar to CD8+ T cells. But unlike B cells and T cells, NK cells do not express specific recognition receptors, but rather they express many regulatory receptors related to their activation and inhibition to recognize “self” and “non-self” components of the body, thereby non-selectively attacking abnormal cells. Activated NK cells release cytotoxic substances through the degranulation pathway to kill tumor cells, while they also secrete chemokines and cytokines to regulate other immune cells [200]. Although NK cells have strong antitumor activity, traditional treatment methods such as radiotherapy/chemotherapy and surgical resection have impaired the function of NK cells: they not only did not eliminate tumor cells, but they also provided them with opportunities for growth and metastasis. Therefore, in order to improve the efficacy of NK cell-based therapies in traditional treatments, various research protocols have been employed to address these challenges, such as adoptive NK cell therapy [201], cytokine therapy [202], bi- and tri-specific killer cell engagers [203], checkpoint blockade [204,205], and oncolytic virus therapy [206].

The emergence of peptide-based nanomaterials has attracted great attention because it can guide the function of NK cells, improve the efficiency of immunotherapy, and have the potential to help NK cell activation, proliferation, and enhancement, and these capabilities are expected to be a turning point in NK cell tumor immunotherapy. Typically, different antibodies or cytokines are loaded together to form a new DDS, and functional peptides are decorated on its surface. This delivery system can simultaneously target both NK cell surface receptors and tumor cell surface antigens, allowing NK cells and tumor cells specificity to bind tightly, where in NK cells can quickly clear tumor cells once activated [176]. The activation of NK cells requires the regulation of different co-stimulatory receptors, so currently used antibodies mainly target the activation or inhibition signal receptors of NK cells. CD16A appears to be the only receptor for IgG Fc fragments to autonomously activate NK cells, and increasing its density can enhance NK cells activity [207]. Monoclonal antibodies are a class of antibodies that target NK cell inhibitory signaling receptors, including killer immunoglobulin-like receptor (KIRs) inhibitors [208] (e.g., IPH2, IPH2201, and IPH2) and ICIs [209,210](e.g., anti-Pd-L1, anti-CD96, and anti-TIGIT). NK cell receptors include CD16, NKp30, NKp46, and NKG2D [211]. Common antigens include CD19, HeR2, and CD33 [212]. As a result, there are several combinations of DDSs based on these targets.

Park et al. developed a polypeptide-based multifunctional microparticle (MNA) [189]. The surface of the MNAs were modified with a variety of functional biomolecules, including antibodies that stimulated NK cells, by binding to NK cell-activating receptors, which are antibodies that capture cytokines secreted by NK cells and polypeptide sensors that are sensitive to granzyme B. In addition to being a sensor, granzyme B peptide can also be used to stimulate anti-2B4 and anti-NKG2D antibodies of NK cells. When the MNA was exposed to granzyme B, the cleavage site designed on the granzyme B peptide was triggered, and the cleavage occurred, allowing a fluorophore (Alexa 488) to be released from the MNAs. The results of the NK cell viability measurement showed that the PE fluorescence intensity of IFN-γ-treated MNAs increased with the increase in IFN-γconcentration, while the fluorescence signal of Alexa 488 decreased with the increase of granzyme B concentration. When MNAs were mixed with NK cells in the same proportion, the sensor signals significantly decreased, and the IFN-γ signal significantly increased. These results suggest that MNAs were able to stimulate NK cells to release IFN-γ and granzyme B as expected to sense the function of NK cells.

Wei et al. synthesized a self-assembled selenopeptide nanoparticle (SeP) that was able to activate NK cells through the oxidative metabolites of selenopeptides to enhance tumor chemoimmunotherapy [213]. The selenium peptide molecular structure includes the RGD peptide, the MMP-2 enzyme-cleavable linker peptide (PLGVR), and the N-terminal Se-dodecyl-selenocysteine double-chain tail with an ROS response (Sec(Dod)_2_K). Through enzyme-induced size reduction and its ROS-driven de-selenation advantage, this selenopeptide could deliver DOX to solid tumors and further activate NK cells in a programmed manner. It is important to note that both in vitro and in vivo studies demonstrated that the chemotherapy induced by DOX and the immunotherapy induced by selenium peptides were mutually promoting, and they synergistically enhanced the antitumor effect, resulting in a tumor suppression rate of 86% (tumor volume less than 200mm^2^ within 29 days). Meanwhile, these selenium peptide nanomedicines exhibited minimal hematological, hepatic, and renal toxicity characteristics.

## 4. Peptide Drug Delivery Vehicles

The above two parts mainly focus on polypeptides as functional sequences to make the carrier/system more intelligent; the following part summarizes polypeptides as nanocarriers to deliver anticancer drugs.

It has been reported that polypeptides, such as PLL and PGA, have been considered as biodegradable candidates for several formulations in clinical trials. Typically, drugs are loaded into peptide nanomaterials through chemical conjugation and physical encapsulation. Chemical conjugation refers to the process of forming a covalent bond between a peptide and a drug molecule through a chemical reaction. The active groups that undergo chemical reactions generally include carboxyl groups, amino groups, hydroxyl groups, sulfonic acid groups, etc. Physical encapsulation refers to the process by which a peptide forms a non-covalent bond with a drug under kinetic or thermodynamic conditions by generating molecular forces such as electrostatic interactions, hydrophobic interactions, hydrogen bonds, and van der Waals forces. For chemical coupling, polypeptides have high drug loading capacity and good stability due to the presence of a large number of active side chain groups. However, this method is complex and difficult to synthesize, and it may introduce potential chemical toxicity and low release in vivo. In contrast, forming DDSs using non-covalent bonds is less stable, but it can ensure full drug release while enhancing antitumor effects.

In an example of chemical coupling, Xiong et al. synthesized a γ-PGA-citric acid (γ-PGA-CA) material with sustained release properties for the delivery of cisplatin (CDDP) [214]. CDDP was conjugated by its own chloride ion to replace the carboxyl hydrogen ion in CA, and the coupling efficiency was high (65%). The system released drugs in bursts within the first 8 h and cumulatively released 50% in 48 h. In vitro and in vivo studies showed that nanoconjugates were significantly less toxic and had a higher tolerated dose compared to unconjugated CDDP. In ICR mice, the area under the curve and systemic clearance of the nanoconjugates were 9-fold and 1/20 of the values for the CDDP, respectively. In addition, the nanoconjugates gradually accumulated at the tumor site after injection and exhibited longer residence times and higher antitumor activity.

**Figure 7:**
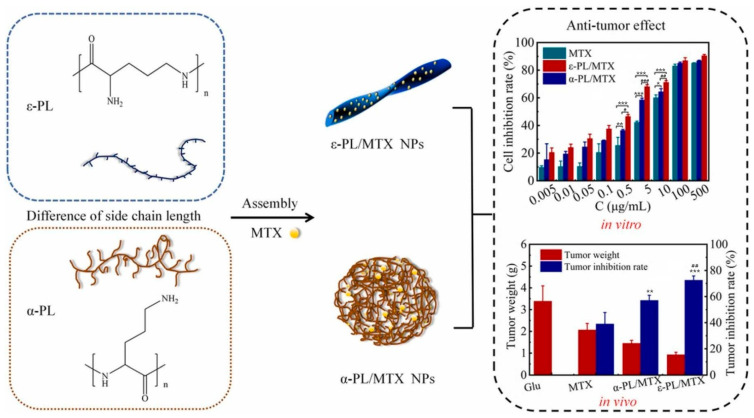
Comparison of the antitumor effects of ε-PL/MTX NPs and α-PL/MTX NPs NPs in vitro and in vivo. DDSs were constructed using two isomers, ε-polylysine (ε-PL) and α-polylysine (α-PL), as carriers to encapsulate methotrexate (MTX). The two NPs obtained not only differed in morphology but also exhibited significantly different antitumor effects in vitro and in vivo. In vitro: 4T1 cells incubated with MTX solution and NPs for 48 h, n = 5. * *p* < 0.05, ** *p* < 0.01, *** *p* < 0.001 vs. MTX; ^#^ *p* < 0.05, ^##^ *p* < 0.01, ^###^ *p* < 0.001 vs. α-PL/MTX NPs; in vivo: tumor weight and tumor inhibition rate calculated from tumor weight, n = 6. ** *p* < 0.01, *** *p* < 0.001 vs. MTX group; ^##^ *p* < 0.05 vs. α-PL/MTX NPs. Reproduced from Reference [217] with permission. Copyright © 2023 Elsevier Masson SAS. All rights reserved.

Electrostatic interaction is a mutual attraction between positively charged and negatively charged groups. With the help of this force, Yu et al. successfully prepared peptide DDSs, namely, polylysine NPs (PLL-MTX NPs) [215]. The preparation of PLL-MTX NPs combined grinding and high-pressure homogenization. Using the friction, impact, and shear forces generated by the grinding medium, the hydrophobic MTX material was crushed into small particles. A strong electrostatic interaction occurred between the carboxyl groups of these small particles and the amine groups of the PLL to form stable drug-loaded NPs, which were then homogenized at high pressure to make them finer and more homogeneous. These NPs exhibited good stability and uniformity in aqueous solutions. PLL-MTX NPs exhibited a higher drug loading content of 58.90% and a smaller size of 113.70 nm compared to amphiphilic NPs (PEG-PLL/MTX NPs). In addition, PLL-MTX NPs were spiral nanorod-like in shape, while PEG-PLL/MTX NPs were nanosheets in shape. According to relevant studies, the rod-like shape should exhibit better activity [216]. In this experiment, the antitumor activity of PLL-MTX NPs was 1.9 times and 1.2 times that of MTX and amphiphilic NPs, respectively. To further research the secondary structure of PLL, the authors used circular dichroism to detect and analyze the conformation of PLL and PLL/MTX NPs, and the characteristic peaks showed that both PLL and PLL-MTX NPs were α helixes. Moreover, they further studied the influence of the secondary structure of the PLL on the antitumor effects [217]. Two different forms of PLL, ε-PLL and α-PLL, were utilized to create DDSs (Figure 7). The ε-PLL/MTX NPs, which had a helical nanorod shape, exhibited small particle size and high drug loading content. In both in vivo and in vitro experiments, the anticancer effect of ε-PLL/MTX NPs was found to be 1.3-fold and 2.6-fold stronger than that of α-PLL/MTX NPs, respectively. These findings suggest that ε-PLL has great potential as a drug carrier for clinical applications.

In hydrophobic interactions, the hydrophobic moiety of the peptide is able to trap the hydrophobic agent through hydrophobic interactions, forming the core of the NPs, while the hydrophilic moiety acts as a shell to improve its water solubility [218,219]. Guo et al. used two pH-sensitive isomers, α-PGA and γ-PGA, as carriers to prepare DDSs [219]. Due to the differences in the hydrophilic/hydrophobic volume ratio, steric hindrance, and surface charge of these two isomers, they had different trapping abilities for hydrophobic drugs. As a result, the chance of α-PGA interacting with drugs was significantly higher. Based on α-PGA, uniform and stable NPs could be obtained with high drug loading (66.2%). In a weakly acidic environment, α-PGA enables a continuous and slow release of DOX, and the cumulative release rate is higher than that in the neutral environment. α-PGA/DOX NPs exhibited several advantages over free DOX, such as lower IC_50_ values, higher tumor suppression rates (67.4%), and lower systemic and cardiotoxic effects.

Additionally, van der Waals forces and hydrogen bonds are used to prepare NPs. Zhang et al. prepared two amphiphilic PGA NPs (α-PGA-F and γ-PGA-F NPs) encapsulated with OVA to induce potent cellular immune responses [220]. Amphiphilic copolymers were synthesized by grafting phenylalanine ethyl esters, and their grafting degrees were 45% (α-PGA-F) and 63% (γ-PGA-F), respectively. In a water solution, when the polymers self-assembled themselves, the OVA could be loaded into them. The maximum encapsulation efficiency of γ-PGA-F and α-PGA-F NPs was about 88%, with no significant difference. However, only α-PGA NPs could induce an α-helical secondary structure under acidic conditions, which could accelerate membrane fusion and antigen lysosomal escape. Furthermore, APCs treated with OVA@α-PGA-F NPs exhibited increased secretion of inflammatory cytokines, along with increased expression of MHC I and CD80.

From the perspective of drug loading methods, whether chemical coupling or physical encapsulation, they all have their own advantages and disadvantages. If there are no specific requirements, peptide nanomaterials are suitable for most drugs, such as DOX, PTX, CDDP, curcumin, etc. For chemical conjugation, those drugs with abundant active groups are more suitable to be entrapped by peptides, which can be conjugated with nanocarriers. For physical entrapment, those drugs with high hydrophobicity, a strong surface charge, and a conjugate structure are more suitable to be entrapped via hydrophobic interactions, electrostatic interactions, and π–π stacking interactions.

## 5. Summary and Outlook

The main components of the TME include malignant tumor cells, immune cells (e.g., T cells and DCs), CAFs, tumor extracellular matrices, and blood vessels. These immune cells and related stromal components, which are recruited and activated by tumor cells, form a tumor-suppressive inflammatory environment that hinders tumor development in the early stages of tumorigenesis or growth. However, after a sustained immune response, the immune cells within the microenvironment experience exhaustion or restructuring, rendering them incapable of carrying out their usual functions and potentially contributing to the malignant features of tumors, resulting in an immunosuppressive microenvironment [221]. Due to the complexity of the TME, it is difficult to achieve a cure for tumors with existing treatments, and the 5-year survival rate of patients is low [222]. Most of the drugs for chemotherapy and immunotherapy have the problems regarding low bioavailability, water solubility, and poor stability, and the use of nanodelivery systems can solve these shortcomings. The nanodelivery system has many advantages for the application of peptides. In addition to being used as a carrier to load fat-soluble drugs, peptide materials can also provide active targeting, permeability, and stimulus responsiveness for the delivery system, as well as enhance the antitumor effect of the delivery system. For different tumor cells and immune cells, different peptide chain sequences can be selected to achieve targeted delivery.

The introduction of peptide materials has partially addressed certain challenges in drug delivery; however, there are still constraints, and they have not yet received clinical approval. First, the specificity and affinity of the peptides are unsatisfactory, leading to inadequate accumulation of the drug at the tumor site. Therefore, it is necessary to screen suitable candidate peptides from large peptide libraries, but the screening method is also a key issue. Traditional selection methods such as combinatorial chemistry library screening (including manual separation, identification, resynthesis, and affinity detection) and phage display technology are inefficient and cumbersome. To solve these problems, artificial intelligence (AI) technology provides a possible method. AI technology can significantly accelerate the discovery and exploration process of candidate peptides through efficient algorithms and big data analysis. At the same time, AI can also help scientists accurately search and screen massive peptide libraries, reducing the total amount of peptide libraries to the most critical and effective parts, saving time and resource costs. The peptide libraries generated through AI would accelerate peptide drug development, provide more information about peptide structure and function, and accelerate personalized therapy design. So far, many high-throughput chips and degenerate peptide libraries have been developed [34]. For example, Cesar de la Fuente-Nunez’s team used elaborate algorithms to mine 2603 cryptic antimicrobial peptides in the human proteome based on their physicochemical properties [223]. Wang et al. developed a high-throughput screening platform based on microfluidic chips [224]. The platform not only has a fast-screening speed but also enables real-time, online, label-free detection, and it innovatively realizes simultaneous qualitative (in situ sequencing) and quantitative (in situ affinity characterization) analyses of two sets of arrays, which are incomparable to traditional combined library screening methods. Chevalier et al. developed a computer modeling platform called “Rosetta” that reduced the total peptide library capacity to 22,660 and designed thousands of non-naturally derived peptides of about 40 amino acids in length [225]. Rosetta [226] (specification of the XML format can be found at http://www.w3.org/TR/2000/REC-xml-20001006 (last accessed on 6 September 2024)) modeling software predicts that these peptides can not only bind tightly to molecular targets, but they can also inhibit the normal function of the target protein. The library of 22,660 peptides and unnaturally derived peptides will provide broader possibilities for the search for novel peptide drugs. These peptide drugs may play an important role in the treatment of cancer, immune system-related diseases, and nervous system-related diseases. Through in-depth analysis and experimental verification of this huge peptide library, it is expected to find novel drug candidate compounds with more efficient and less toxic side effects, thereby enhancing the clinical potential of peptide drugs and bringing more treatment options to patients. Second, systemically administered peptide-based nanodrugs are easily cleared by the liver sinusoidal endothelium, causing a substantial decrease in the delivery efficiency of nanomedicines into target tissues. To prolong the circulation time in the blood and enhance the accumulate rate of nanomedicines in tumor tissue, “PEGylation” is a strategy that has been studied widely [227,228,229]. After coating PEG chains, the interaction between NPs and plasma proteins could be decreased, resulting in a prolonged systemic circulation time [229,230]. In addition, the “stealthy” property of the PEG could protect NPs and decrease the rapid clearance by reticuloendothelial system in the liver, promoting the delivery efficiency of nanomedicines into the target tissues [231]. Dirisala et al. demonstrated that two-arm PEG-coupled oligopeptides can selective and transient stealth coating liver scavenger cells, which avoids the rapid capture of nanomaterials and relocates them to target tumor tissue [231]. This research reveals that PEGlyation is an effective strategy to improve drug distribution in vivo. Gref et al. prepared a kind of PEG-coated biodegradable nanosphere [232]. Experimental results showed that 66% of uncoated PLGA nanospheres were detected in the liver, while only 5% of nanospheres were observed in the blood. On the contrary, 17% of coated PLGA nanospheres were in the liver, and more than 40% of nanospheres existed in the blood after coating PEG chains. As expected, the stealthy property of the PEG could decrease the non-specific clearance by the liver.

In conclusion, peptides as naturally occurring substances show great potential for use in tumor immunotherapy, and the development of new screening methods and construction of reasonable functional molecules are effective ways.

## Figures and Tables

**Table 1:** List of some peptide drugs approved for clinical use.

Name	Date of Approval	Specification	Indications	Ref.
Eptifibatide injection	12 March 2019	10 mL: 20 mg	For patients with acute coronary syndromes (unstable angina/non-ST-elevation myocardial infarction), including those receiving medical therapy and those undergoing percutaneous coronary intervention (PCI).	[41,42,43]
Liraglutide injection	28 March 2023	3 mL: 18 mg (prefilled injection pen)	It is used to treat type 2 diabetes mellitus in adults.	[44,45,46]
Semaglutide	December 2020	Tablets: 3 mg × 100 capsules, 7 mg × 100 capsules, 14 mg × 100 capsules; Injections: 1.34 mg/mL, 1.5 mL (prefilled injection pen)		[46,47,48]
Lixisenatide injection	28 July 2016	10 μg dose injection pen (green): 0.05 mg/mL, 3 mL/piece, single injection dose 10 μg (0.2 mL)		[49]
Tirzepatide injection	May 2022	0.5 mL injection; 2.5, 5, 7.5, 10, 12.5, 15 mg		[46,50,51]
Pramlintide	17 March 2005	3 mg/5 mL	It is used for the treatment of type 1 and type 2diabetes mellitus in adults.	[52,53,54,55]
Leuprorelin Acetate Microspheres for Injection	20 June 2011	11.25 mg (as leuprolide acetate)	Prostate cancer, premenopausal breast cancer, central precocious puberty	[56,57]

**Table 2:** List of advantages and disadvantages of polypeptides.

Peptide/Sequence	Receptor	Advantages	Disadvantages	Ref.
RGD	Integrin	Targeting, high affinity	Lack of target specificity, poor permeability to solid tumors or tissues, short half-life, poor stability in the biological environment, heterogeneity of integrin expression	[41,59,60]
NGR	CD13	Tumor-targeting, high affinity	Poor permeability to solid tumors or tissues, lack of target specificity	[61,62,63]
T7 (HAIYPRH)	TfR	Tumor-targeting, Blood-brain barrier (BBB) penetration	Poor permeability to solid tumors or tissues	[64]
iRGD (CRGDKGPDC)	Integrin	High affinity, tumor-targeting, permeability	Lack of target specificity	[65,66]
iNGR (CRNGRGPDC)	CD13	Tumor-targeting and permeability	Lack of target specificity	[61,62,67]
tLyp-1 (CGNKRTR)	P32	Tumor-targeting, permeability, high affinity	Tumor penetration is limited	[68]
RLW (RLWMRWYSPRTRAYG)	N/A	High permeability and strong targeting.	To be assessed	[69,70]
F3 (CKDEPQRRSARLSAKPAPPKPEPKPKKAPAKK)	Nucleolin	Targeting, high affinity	Tumor penetration is limited	[68,71,72]
TAT (YGRKKRRQRRR)	Nuclear mucin	Good targeting	Tumor penetration is limited	[73,74]
(KlaKlaK)_2_	Mitochondria	Targeting, anticancer activity	Non-permeability, toxic to normal tissues or cells	[75,76]

**Table 3:** List of responsive peptide-based materials.

Type	Carrier	Appearance	Tumor Model	Ref.
pH-responsive	Lip-HE-R6	Oval granules	4T1	[139]
pH-responsive	1-RGDH	Nanofibers	A549	[140]
pH-responsive	PLGA-b-PPO-b-PLGA	Vesicles—spherical micelles	N/A	[141]
pH-responsive	PpIX-Ahx-AEAEAKAKAEAEAKAK	Spherical NPs—rod-like NPs	HeLa	[142]
pH-responsive	C16-VVAEEE	Nanofibers	MvE, A431, HeLa, HEK293	[143]
pH-responsive	FER-8	Nanofibers	HepG2	[144]
MMPs-responsive	MePEG-Peptide-Tri-CL	Star-shaped NPs	A549	[145]
MMPs-responsive	Nap-FFGPLGLARKRK	Nanofibers	HepG2	[146]
Cathepsin B-responsive	AuNR@FrFK-GFLG-EK	Gold nanorods	4T1	[147]
Cathepsin B-responsive	HCPT-FF-GFLG-EEYSA	Nanofibers	T24	[148]
Cathepsin B-responsive	HCy-Cit-Val, HCy-Gly-Leu-Phe-Gly	Nanoprobes	HeLa	[149]
ENTK-responsive	Mito-Flag-MNP	Nanomicelles–nanofibers	HeLa	[150]
Caspase-responsive	Nap-GFFpYDEVD-AFC, Nap-GFFpYIETD-AFC	Nanofibers	HeLa, MCF-7	[151]
Lysyl oxidase-responsive	Ac-Ala-Ala-Ala-Ala-Ala-Ala-Lys-Lys-NH2	Nanovesicles–nanofibers	BEL-7402	[152]

**Table 4:** List of peptide-based nanocomplexes for tumor immunotherapy.

Nanocomposite	Immune Adjuvant	Target	Tumor Model	Ref.
T7-PEG-TMC-TBC NPs	siRNA, Anti-PD-L1	TfR, PD-1/PD-L1	4T1	[177]
^Super^PDL1^exo^NPs	Anti-PD-L1	PD-1/PD-L1	MC-38	[178]
IR780-M-APP NPs	Anti-PD-L1	PD-1/PD-L1	B16F10	[179]
CLIP6-OVA/CpG NPs	CpG	MHC I, MHC II	B16-OVA	[180]
PLGA NPs	N/A	MHC I	E·G7-OVA	[181]
sHDL	CpG	Anti-PD-1, anti-CTLA-4, APCs	MC-38, B16F10	[182]
B13G-Au NPs	N/A	Dectin-1	P388D1	[183]
C16-K(PpIX)-PEG8-KDEVD-1MT NPs	1MT	IDO	CT26	[184]
RNAi-Au NPs	Anti-VEGF	VEGF mRNA (TAMs)	A549	[185]
M2NP-siCD115	N/A	CSFs-1/CSFs-1R(TAMs)	B16	[186]
ChiP-RS NPs	R848	SHP-2, TAM2	4T1	[187]
161519TriKE	Anti-CD16, IL-15, Anti-CD19	CD16, IL15, CD19 (NK cell)	PBMC	[188]
MNAs	Anti-2B4, Anti-NKG2D	2B4 (CD244), NKG2D (CD314)	K562	[189]
